# Educational games created by medical students in a cultural safety training game jam: a qualitative descriptive study

**DOI:** 10.1186/s12909-022-03875-w

**Published:** 2022-11-22

**Authors:** Juan Pimentel, Paola López, Camilo Correal, Anne Cockcroft, Neil Andersson

**Affiliations:** 1 CIET-PRAM, Department of Family Medicine, McGill University, 5858 Chemin de La Côte-Des-Neiges 3Rd Floor, Suite 300, Montreal, QC H3S 1Z1 Canada; 2 Facultad de Medicina, Universidad de La Sabana, Campus Universitario Puente del Común, 250001 Chía, CP Colombia; 3 Centro de Investigación de Enfermedades Tropicales (CIET), Universidad Autónoma de Guerrero, Calle Pino S/N Colonia El Roble, 39640 Acapulco, Guerrero Mexico

**Keywords:** Cultural safety, Medical education, Colombia, Game jam, Serious games

## Abstract

**Background:**

Cultural safety training, whereby health professionals learn to reflect on their own culture and to respect the cultural identity of patients, could address intercultural tensions in health care. Given the context of their medical education, however, medical students might perceive such training to be dull or even unnecessary. Game jams, collaborative workshops to create and play games, are a potentially engaging learning environment for medical students today. How medical students learn while making games is poorly documented. This study describes the characteristics of educational games created by participants in a cultural safety game jam and the concepts they used to create games.

**Methods:**

As part of a trial**,** 268 Colombian medical students divided into 48 groups participated in an eight-hour game jam to create a prototype of an educational game on cultural safety. In this qualitative descriptive study, we reviewed the description of the games uploaded by participants, including the name, objective, game narrative, rules, rewards, penalties, and pictures. An inductive thematic analysis collated their descriptions.

**Results:**

The game descriptions illustrated the characteristics of the educational games and the aspects of the cultural safety concept that the students used to create games. Medical students situated cultural safety within a continuum with culturally unsafe actions at one end and cultural safety at the other end. Although not familiar with game design, the students designed prototypes of basic educational games including game dynamics, game scenarios, learning objectives, and pedagogical strategies.

**Conclusion:**

The findings of this study could help researchers and educators to understand how medical students learn from game design and the kind of games that game jam participants can create without previous game design skills.

**Supplementary Information:**

The online version contains supplementary material available at 10.1186/s12909-022-03875-w.

## Background

Cultural safety encourages Western-trained health practitioners to examine how their own culture shapes their clinical practice; it proposes they should respect patient worldviews [1]. Lack of cultural safety in health care is linked to stigma and discrimination towards culturally diverse patients [2].

As part of an effort to bridge the cultural divide between Western health services and the cultural preferences of the society, we developed a curriculum segment to promote cultural safety skills of medical students at La Sabana University in multicultural Colombia [3–6]. A challenge facing cultural safety education, however, is that medical students might perceive it as dull or even unnecessary [7].

*Game jams*, collaborative workshops to create and play educational games, have recently shown effectiveness and engaging potential in university-level education [8, 9]. Game jams are a relatively recent phenomenon with the earliest documented in 2002 [10]. These events have been used for purposes beyond game design. Examples include the Health Games Challenge Game Jam, promoted by Michelle Obama in 2010; the Fukushima Game Jam, aimed at assisting after the 2011 earthquake and nuclear disaster [11]; and the utilization of the game jam model as a research co-design method [12]. Through their Sami Game Jam, Laiti and collaborators[13] suggested that game jams could promote self-discovery, reflections on identity, and support the cultural identity of minority groups, such as the Sami people from Finland.

Evidence suggests that people perceive game jams as a fun and engaging method to assimilate new knowledge and skills [9]. Having fun through collaborative learning, with ownership of the material and messages as part of the process rather than as the education outcome, could offer a stark contrast to conventional lecture halls and multiple-choice questions [14]. Game jam learning has received particular attention in Finland, where the national curricula has shifted towards promoting meta skills like learning-to-learn and communication. [15, 16] Finish researchers have commented that game jams could help to develop 21st-century skills [15], which are post-industrial competencies such as metareflexion and epistemic flexibility, that prioritize adaptation and innovation rather than the acquisition of fixed information. [17, 18]

In 2019, a randomised controlled trial (RCT) tested whether medical student participation in a game jam on cultural safety is more effective than conventional education in changing self-reported intended patient-oriented behavior and confidence in transcultural skills [19]. A total of 531 students participated and game jam participants reported higher confidence in their capacity to execute culturally safe behaviors [20] and a transformative impact of cultural safety training on a results chain from conscious knowledge through to action [21]. This was the first published medical education research to explore the potential of game jam learning in medical training. This study describes here the characteristics of the educational games created by the game jam participants and the aspects of the cultural safety concept that the students used to create their games.

## Methods

### Study design and research question

This qualitative descriptive study used inductive thematic analysis. Our research questions were: what are the characteristics of the educational games created by the medical students, and what aspects of the cultural safety concept did the students use to make games? Our manuscript adheres to the Standards for Reporting Qualitative Research (Additional file 1) [22].

### Setting and participants

We conducted the game jam at *La Sabana* University in Chia, located 20 min from the Colombian capital Bogota. *La Sabana* is a private university with close to 9,000 undergraduate students, including 956 medical students and 256 medical interns [23].

The inclusion criteria for this study were being a medical student or medical intern at any level of training and giving written informed consent. The exclusion criteria were being underage or not wanting to participate in the study. We contacted the medical students and medical interns using *La Sabana* University’s mailing lists and e-mailed invitations for voluntary participation in the project. 268 students participated in the eight-hour game jam.

The game jam aimed to create a prototype of an educational game on cultural safety. The activity included: (a) preliminary lecture on cultural safety and game design; (b) game building session where groups of participants created educational games about cultural safety; and (c) play-test session in which participants played and learned from each other’s games. The lecture was based on our co-designed curriculum[3] and provided key elements of cultural safety, including (a) the principles of cultural safety; (b) consequences of cultural tensions in health care; (c) self-awareness; (d) Colombian cultural health practices; and (e) respect for patients who use traditional and cultural health practices.

### Data collection

**Fig. 1:**
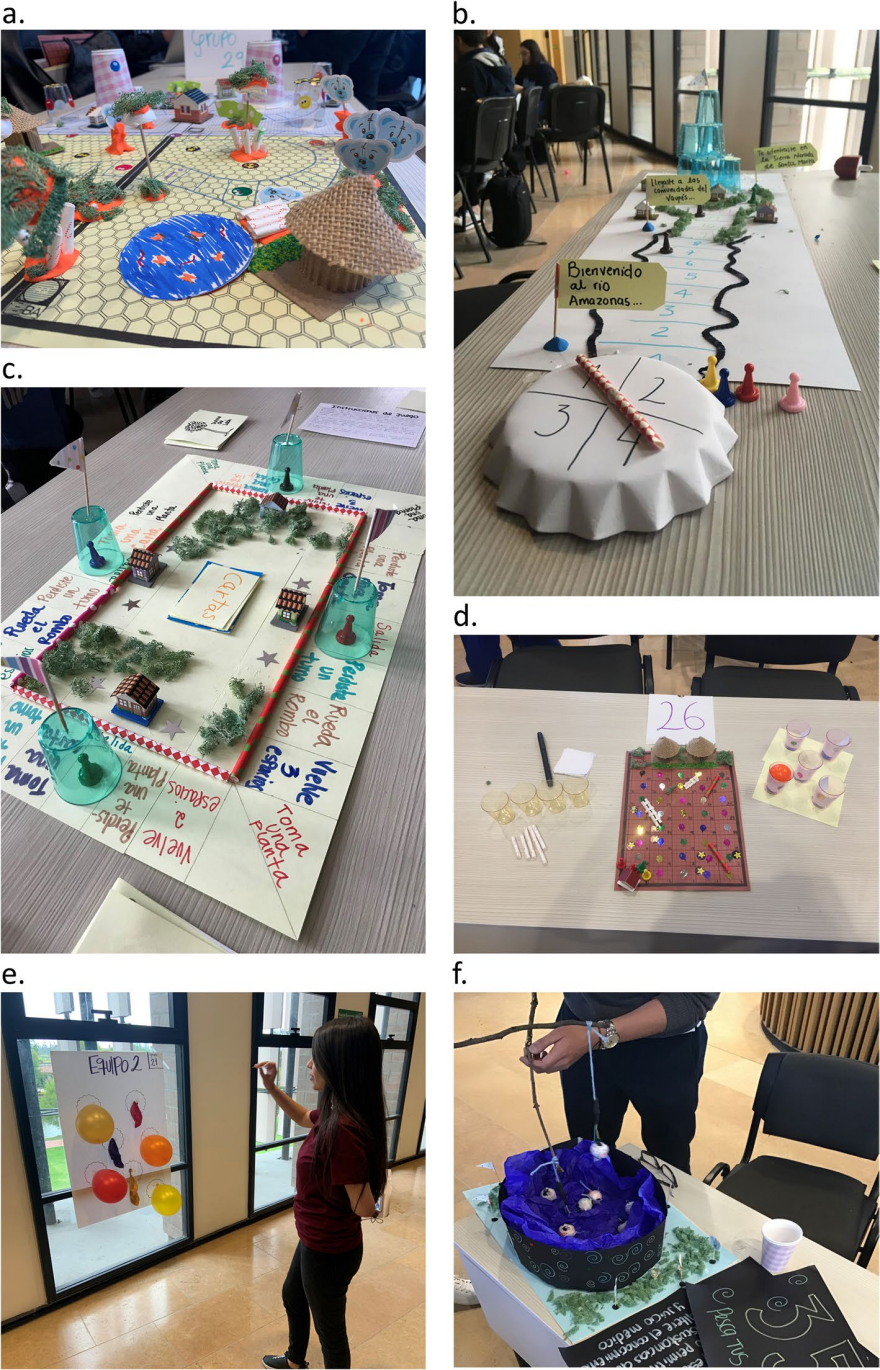
Prototypes of educational games about cultural safety created by the medical students. The students created board games based on, for example, snakes and ladders (**a**, and **d**), carcasonne amazonas (**b**) and monopoly (**c**). They also created games other than board games (**e** and **f**)

Each of the 48 groups of 4–6 students designed a low-tech prototype of an educational game about cultural safety (Fig. 1). This study used a pre-defined format in Google forms and asked each group to upload a description of their game including the name, objective, an explanation of the game, and pictures.

### Data processing and analysis

We used Dedoose 9.0.17 to support our analysis. Two researchers (JP and PL) individually coded the descriptions of the games following an inductive thematic analysis approach, “a method for identifying, analysing and reporting patterns [themes] within data” [24]. An inductive approach means that the themes, which are closely linked to the data, are identified from the ‘bottom-up’ [25]. JP and PL later compared their coding and, by consensus, decided on themes and subthemes. The two researchers held six two-hour meetings to carry out the qualitative analysis.

### Rigour

This study adhered to the strategies for ensuring trustworthiness in qualitative research suggested by Patton[26] and Shenton [27]. We increased credibility by using validated research methods such as standard inductive thematic analysis to process the data. The study enhanced dependability by adhering to the Standards for Reporting Qualitative Research. We increased confirmability by disclosing the background of the researchers directly involved in the data analysis, as well as by recognizing the limitations of the study. JP, an MD with an MSc in Epidemiology and a PhD in Family Medicine and Primary Care, guided PL during the analysis phase. PL was a fifth-year medical student with previous experience in qualitative research and cultural safety when this article was written.

### Ethical approval

The Institutional Review Board of the McGill’s Faculty of Medicine (approval number A05-B37-17B) and the Sub-committee for Research of the Faculty of Medicine at *La Sabana* University (approval number 445) provided ethical clearance for this study. All participants signed informed consent before proceeding with any research activity.

## Results

Table 1 provides a description of the sociodemographic characteristics of the game jam participants. Most of them were middle-class females, born and living in Bogota, attending their clinical rotations.

**Table 1:** Baseline sociodemographic characteristics of the game jam participants (*n* = 268)

	**n (%)**
**Sex**	
Female	182 (67.9)
Male	85 (31.7)
Prefer not to say it	1 (0.4)
**Place of birth**	
Bogota	146 (54.5)
Colombia, another city	96 (35.8)
Venezuela	19 (7.1)
Another country	7 (2.6)
**Place of residency**	
Bogotá	169 (63.1)
Colombia, another city	99 (36.9)
**Socioeconomic level**	
One – lowest	5 (1.9)
Two	12 (4.5)
Three	55 (20.5)
Four	85 (31.7)
Five	67 (25)
Six – highest	34 (12.7)
Prefer not to say	2 (0.7)
Don’t know	8 (3)
**Education level**	
II and III semester (preclinical)	62 (23.1)
VI to XI (Clinical)	191 (71.3)
Medical intern	15 (5.6)
**Age in years**	
Min	18
Max	31
Mean (SD)	20.9 (1.9)

Our study identified four themes and 17 subthemes describing the characteristics of the educational games, and four themes and 12 subthemes describing the cultural safety aspects that the students used to create games (Table 2).

**Table 2:** Themes and subthemes describing the cultural safety aspects that the students used to create games and the characteristics of the educational games

Aspects of the cultural safety concept used by the students	
**Theme**	**Subtheme**
Culturally unsafe actions	Aggression
	Consequences of culturally unsafe actions
	Imposition of modern medicine
Self-awareness	Bias/beliefs regarding traditional medicine
	Evidence-based medicine
Cultural safety	Benefits
	Continuum
	Principles
Characteristics of traditional and cultural health practices	Providers
	Difference from other types of medicine
	Traditional illnesses and remedies
	Health of women
**Characteristics of the educational games**	
Learning objective	Knowledge
	Attitudes
	Clinical practice
Scenarios	Geographical places
	Intercultural clash
	Diseases
	Medical specialties
	Compulsory social service
Game dynamics	Penalties
	Game characters
	Rewards/incentives
	Game resources
	Rules
	Types of game
Pedagogical strategy	Previous knowledge
	Case resolution
	Roleplay

### Aspects of the cultural safety concept used by the students

The students situated their game within a continuum or steps to reach cultural safety:

*“The player will have to go over the steps towards cultural safety” (group 33)*

The journey usually starts with the player situated on the opposite end of cultural safety: culturally unsafe actions. This is comprised of aggression towards cultural minorities and imposition of modern medicine, which entails consequences for patients and health professionals:

*“Our game is based on “Clue” but is adapted to follow cultural safety. First, the culprit of engaging in culturally unsafe actions is sought. […] culturally unsafe actions are, for example, judging, scolding, discriminating against, etc.” (group 4)*

*“Culturally unsafe actions affect the doctor-patient relationship and our professional practice, thus creating a distance between our patients and their communities.” (group 34)*

The games emphasized that, after reflecting on culturally unsafe actions and their consequences, the next step is self-reflection. This includes learning about bias and misconceptions about traditional medicine, and the role of evidence-based medicine in preventing students exploring other types of medicine:

*“The objective of the game is to make the players reflect on whether they have or have had bias. It fosters self-reflection about how you see other practices.” (group 17)*

The students also highlighted the need to learn, through the game, about the main characteristics of traditional and cultural health practices in Colombia. This includes providers (bonesetters, midwives, healers), traditional illnesses and remedies, and a special focus on women´s health.

*“The game will teach about traditional medicine and how to face different situations with patients who practice traditional medicine. It will also help to identify our shortcomings as practitioners of modern medicine” (group 9)*

*“One of the characters of the game, a traditional medicine user, has stopped following the traditional advice to promote women’s health that has been always used in the family, like avoiding walking barefoot, drinking warm plants tea, and bathing with hot water when menstruating." (group 44)*

Finally, the students highlighted the need for the games to teach about the benefits and principles of cultural safety. The benefits included improving the doctor-patient relationship and adherence to treatment, a better quality of health services, and positive health outcomes.

*“The only way to get to heaven is by rescuing the core values of cultural safety and its benefits in terms of adherence to treatment and continuity of care.” (group 9)*

*“The objective of the game is to ensure that the player learns the importance of cultural safety in clinical practice, so that quality of care and health outcomes improve. But above all, to learn to be respectful towards the patient, thus reducing the gap related to cultural differences.” (group 19)*

### Characteristics of the educational games

The students proposed different learning objectives such as improving the knowledge, attitudes, and skills related to cultural safety:

*“The aim [of the game] is to provide, in a fun way, knowledge about the steps to reach cultural safety” (group 4)*

*“Roleplay in which decisions must be made to show assertive communication, respect for traditional medicine users, and potential integration with Western medicine.” (group 14)*

Students used several scenarios to describe the place and context in which their game takes place. This includes rural areas, as well as situations of intercultural clash or specific clinical cases.

*“An experience of visiting different regions, and with them their cultures” (group 10)*

*“Our game teaches the factors that negatively affect the doctor-patient relationship in the context of a culture clash.” (group 46)*

*“In pediatrics, mothers generally use traditional remedies for common diseases such as diarrhea, nausea, constipation, common cold, and on many occasions, we as doctors do not understand it and prohibit these practices.” (group 22)*

The students also used the compulsory social service which new doctors in Colombia must undertake when they finish medical school, as a potential game scenario:

*“A group of recently graduated physicians come to undertake their compulsory social service year in the village of Kunak. However, they will not be so welcome because they do not know the cultural practices of the Kunak people and rather try to impose modern medicine. Kunak will challenge them by testing their knowledge of traditional medicine. If they complete the mission and win, they can stay and work with the community. If they do not win, they will be expelled from it.” (group 10)*

The students described a broad range of game dynamics, such as penalties, game characters, rewards, rules, and types of game. The game dynamics are described in Table 3.

**Table 3:** Game dynamics used by the game jam participants

**Game dynamic**	**Description**
Penalties	Jail, punishment, community expulsion, penitence, moving back squares, hell
Game characters	Medical students, interns, residents, general practitioners, specialists, educators, leaders
Rewards/incentives	Lives, points, progressing in the game, stars, game money, rewards, heaven, crowns
Game resources	Clues, cards, tokens, dice, medicinal plants, symbols, marbles, air balloons
Rules	Fish the fish, grab medicinal plants, score in goal, build structures, answer questions, connect words, spin the roulette, throw the dice, move the tokens, press the button, collect objects, pop the air balloon
Types of game	Beer pong, snakes and ladders, mini soccer, clue, minigolf, monopoly, pachisi, simulation

The students used three main types of pedagogical approaches embedded in their games: use of previous knowledge, case resolution, and role play:

*“The game uses prior knowledge about cultural practices that the students have experienced or learned in the past.” (group 43)*

*“The player rolls two dice, and they advance on the board. If they fall in a box with a sad face, they must take out a card with a description of a problem about cultural tensions, they have to solve it.” (group 22)*

## Discussion

Our study identified aspects of the cultural safety approach that the students used to create games and the characteristics of the educational games that they conceived. Overall, the students explored cultural safety by using a step-by-step process, moving from cultural destruction towards culturally safe actions. They used a range of game dynamics based on their prior game experiences and knowledge.

### Cultural safety

The students situated cultural safety within a continuum between culturally unsafe actions and cultural safety. In between, they acknowledged the importance of self-reflection about their own culture and the patient culture, with a focus on the basic characteristics of Colombian traditional and cultural health practices. Our problem–solution structure [6], which starts by surfacing or unearthing culturally unsafe actions, motivated the study participants to learn about cultural safety. The students acknowledged the issue of culturally unsafe actions, which naturally led them to wonder how to prevent or address this issue. Our results suggest that medical students engaged with the practical scheme of our co-designed curriculum [6], which we provided prior to the game jam session.

Authors have highlighted the usefulness of a multi-level or spectrum of standpoints leading up to cultural safety in health care education. Ramsden proposed a dynamic process moving from cultural awareness to cultural sensitivity to cultural safety [28]. Wood and Schwass described a model linking culturally unsafe behaviors and cultural safety [29]. Results of the RCT [20] lead us to believe the communicative action of co-designing an educational game might have had this effect.

Culturally unsafe actions have been defined as "any actions which diminish, demean or disempower the cultural identity and wellbeing of an individual" [30]. Several systematic reviews have reported the effects of stereotypes, prejudices or discrimination against minority groups including lower levels of healthcare-related trust, patient satisfaction, adherence to treatment uptake, and delaying or not seeking healthcare [2, 31, 32]. This information, that proved relevant for the game jam participants, allowed them to understand why learning about cultural safety is relevant.

The Nursing Council of New Zealand highlighted the importance of self-reflection in cultural safety education [33]. Ramsden [34] suggested that students should reflect on their own values and attitudes that influence their clinical practice. Students promoted self-reflection in their games, as a preliminary step before learning about the patient culture. They also included some characteristics of Colombian traditional and cultural health practices [35] in their games, such as traditional illnesses and remedies, and traditional medicine providers.

### Game jam learning

Serious or educational games provide the players with an opportunity to immerse themselves in a risk-free, interactive, and engaging environment. In this context, learners assimilate theoretical knowledge while simultaneously applying the concepts learned [36]. Three systematic reviews confirm the beneficial effects of using serious games in medical education [37–39]. No firm conclusions about the learning potential of game co-creation -such as game jam learning- were reported, and the authors recommended further research on the topic. Our study enhanced the available evidence on game co-creation as a learning intervention in medical education.

Mildner and Mueller described common elements of educational games such as play, rules, storytelling, social factors, and learning [40]. Although not familiar with game design, the students were able to design basic prototypes of educational games including game dynamics, game scenarios, learning objectives, and pedagogical strategies. Most game jam groups used previously known games such as snake and ladders, beer pong, and monopoly to make their games. Our study therefore identified that participants used their prior game-related knowledge and experiences, which supports the idea that even participants without prior game design experience can participate in game jam learning.

Trainees used additional real-life experiences and prior knowledge beyond game design. The teaching method elicited memories of past encounters, for example of culturally unsafe actions in clinical practice, to increase the engagement of medical students. All these processes follow a constructivist approach [41] where students use prior knowledge to assimilate new knowledge. It also fosters communicative learning, which is a key element of transformative learning [42] and critical systems thinking [43].

Our study provides evidence that making games can help medical trainees learn collaboratively, which has been reported outside the medical field [11]. Game jams foster a culture of creativity and learning, play testing, idea sharing, and collaboration between “jammers” [9], thus helping the participant to approach the design of the game both as a student and as an author or game designer. These events support the process of learning-by-doing while enhancing creative thinking and innovation [44]. Aurava et al.[15] studied game jams for three years through extensive fieldwork and reported that game jam learning is a viable training method and participants often report perceived learning and motivation.

The flexible nature of game jams allowed students to adapt their learning experience to their particular needs. For example, students proposed different scenarios or narratives to make their game, including the Colombian medical social service -a compulsory social service for one year to help meet pressing healthcare needs in rural areas- [45]. Many of these professionals were born and raised in large urban settings, therefore experiencing intercultural tensions when interacting with people from rural communities, who often use traditional and cultural health practices [4]. The game jam, therefore, allowed them to use the knowledge they thought was most relevant.

We believe that the game jam learning experience is aligned with Mezirow’s theory of transformative learning and constructivism, as previously suggested [15]. In our study, game jam learning was engaging, participatory, interactive, problem-based, and based on prior knowledge of learners. Additionally, by using our co-designed curriculum on cultural safety [6], which was designed with local traditional medicine users and medical students from Colombia, our game jam learning initiative was adapted to the specific characteristics of local communities. Finally, this type of learning is adapted to the learning needs of the Millennial generation, who have a strong relationship with information and technology [46].

### Limitations

We asked the group representative to provide the description of the game on behalf of the game jam group, meaning that our unit of observation was not individual participants. This approach prevented the exploration of subgroup opinions based on, for example, student gender or training level. Additionally, we provided a thematic description of the games created by our participants rather than an individual description of each game.

A well-recognized limitation in medical education research is its risk of social desirability bias [47]. Medical students could respond with the most socially desirable answers rather than their own point of view. We have reported this bias in past medical education qualitative studies [4, 5]. Additionally, researchers have noted that self-assessments of students learning do not necessarily prove that learning has happened during game jam learning [15, 48].

The qualitative nature of our study did not allow for exploration of how game jam-based cultural safety training differed between subgroups of participants (for example considering age, gender, or socioeconomic status). We explored this, however, through our quantitative assessment [20].

Finally, our study is qualitative, and the results are likely to be influenced by our own values and beliefs, which align with those in the fields of biomedicine, family medicine, and primary care. For example, we might have felt inclined to highlight quotations and themes aligned with principles such as compassionate care, a generalist approach, and interpersonal relationships, which drive family practice [49].

## Conclusions

This study identified the characteristics of the educational games and the aspects of the cultural safety approach that the students used to create games. The learning experience the students had through game jam learning is aligned with theories of transformative learning and constructivism. This information could help researchers and educators to understand the kind of games that game jam participants without game design skills can create in eight hours, the aspects of cultural safety that students can use to make games, and the learning theories they can use to inform game jam learning.

## Supplementary Information

**Additional file 1.** Standards for Reporting Qualitative Research (SRQR).*

## Data Availability

The datasets used and/or analysed during the current study will be available from the corresponding author on reasonable request.

## Abbreviation

RCT: Randomised Controlled Trial
